# Cognitive and emotional factors of the subjective readiness to vaccination against coronavirus in Russia

**DOI:** 10.1192/j.eurpsy.2022.1246

**Published:** 2022-09-01

**Authors:** A. Tkhostov, E. Rasskazova, O. Tikhomandritskaya

**Affiliations:** 1 Moscow State University, Clinical Psychology, Mokhovaja, Russian Federation; 2 Mental Health Research Center, Medical Psychology, Moscow, Russian Federation; 3 Moscow State University, Clinical Psychology, Moscow, Russian Federation

**Keywords:** vaccination, readiness, psychological factors

## Abstract

**Introduction:**

Low vaccination rate against coronavirus in Russia demands for studies of psychological factors affecting decision to vaccinate. Readiness for vaccination is related to perceptions of risk, concerns and trust in the source of the recommendations (Chung, Thone, Kwon, 2021, Flanagan et al., 2020).

**Objectives:**

To study the subjective readiness for vaccination against coronavirus and its relationship with pandemic anxiety and attitudes towards vaccination.

**Methods:**

525 people aged 18 to 65 appraised their readiness to vaccination (Cronbach’s alpha .89-.90), filled out Anxiety Regarding Pandemic Scale (Tkhostov, Rasskazova, 2020), modified version Beliefs About Medication Questionnaire (Horne, 2002) that was reformulated to measure beliefs about vaccination in December 2020.

**Results:**

13.2% -17.0% participants reported readiness to be vaccinated. Low readiness rate was due to doubts and mistrust (59.0% -60.4%). Having more friends experienced coronavirus as well as severe or fatal cases of coronavirus illness among personal acquaintances were associated with higher rates of pandemic anxiety but not readiness to vaccinate. Readiness to vaccinate asap was predicted by belief in the effectiveness and lower concern about vaccination (R²=34,6%) and anxiety regarding risks and side effects of the vaccination (ΔR²=1,5%). Decision to refuse was predicted by belief that there are better alternatives of prophylaxis, doubts in effectiveness and concerns about necessity (R²=56,0%).

**Conclusions:**

Decision to vaccinate is based both on cognitive confidence in the importance and effectiveness of vaccination, and on less pronounced anxiety about risks and side effects. Research is supported by the Russian Foundation for Basic Research, project No. 20-04-60072.

**Disclosure:**

Research is supported by the Russian Foundation for Basic Research, project No. 20-04-60072.

